# Identification of immune subtypes to guide immunotherapy and targeted therapy in clear cell renal cell carcinoma

**DOI:** 10.18632/aging.204252

**Published:** 2022-09-01

**Authors:** Chen Xu, Yang Li, Wei Su, Zhenfan Wang, Zheng Ma, Lei Zhou, Yongqiang Zhou, Jianchun Chen, Minjun Jiang, Ming Liu

**Affiliations:** 1 Department of Urology, Suzhou Ninth People’s Hospital, Soochow University, Suzhou 215000, China; 2 Department of Urology, Shanghai Pudong Hospital, Fudan University Pudong Medical Center, Huinan Town, Pudong, Shanghai 201399, China; 3 Department of Medical Oncology, Fudan University Shanghai Cancer Center, Shanghai 200032, China; 4 The State Key Laboratory of Pharmaceutical Biotechnology, Department of Hematology, The Affiliated Drum Tower Hospital of Nanjing University Medical School, China-Australia Institute of Translational Medicine, School of Life Sciences, Nanjing University, Nanjing 210023, China

**Keywords:** clear cell renal cell carcinoma, immune subtype, tumor immune microenvironment, immunotherapy

## Abstract

Accumulating pieces of evidence suggested that immunotypes may indicate the overall immune landscape in the tumor microenvironment, which were closely related to therapeutic response. The purpose of this study was to classify and define the immune subtypes of clear cell renal cell carcinoma (ccRCC), so as to authenticate the potential immune subtypes that respond to immunotherapy. Transcriptome expression profile and mutation profile data of ccRCC, as well as clinical characteristics used in this study were obtained from TCGA database. There were significant differences in the infiltration of immune cells, immune checkpoints, and antigens between ccRCC and para-cancerous tissues. According to immune components, patients with ccRCC were divided into three immune subtypes, with different clinical and molecular characteristics. Compared with other subtypes, IS2 showed cold immune phenotype, and was associated with better survival. IS1 represented complex immune populations and was associated with poor overall survival (OS) and progression free survival (PFS). Further analysis indicated that expression of immune checkpoints also differed among the three subtypes, and was abnormally up-regulated in IS3. Pathway enrichment analysis indicated that the mTOR signaling pathway was abnormally enriched in IS3, while the TGF_BETA, ANGIOGENESIS and receptor tyrosine kinase signaling pathways were abnormally enriched in IS2. Furthermore, there was an abnormal enrichment of the epithelial-to-mesenchymal transition (EMT) signaling pathway in IS1, which may be associated with a higher rate of metastasis. Finally, SCG2 was screened as a specific antigen of ccRCC, which was not only related to poor prognosis, but also significantly associated with immune cells and immune checkpoints. In conclusion, the immune subtypes of ccRCC may provide new insights into the tumor biology and the precise clinical management of this disease.

## INTRODUCTION

Renal cell carcinoma (RCC) originates from the renal tubular epithelial system and is the most common malignant tumor of the urinary system, accounting for approximately 80–90% of malignant renal tumors. In the United States, RCC ranked the eighth most common malignant tumor, accounting for 4.2% of novel diagnoses [1, 2]. Clear cell renal cell carcinoma (ccRCC) was the most common histological type of RCC. Additionally, ccRCC caused approximately 175,000 deaths each year, and approximately 30–35% of patients with ccRCC undergoing surgery developed distant metastases [3]. Although localized ccRCC may be cured by surgical resection, the probability of local recurrence or distant metastasis within 5 years was approximately 30% [4]. Due to the lack of sensitivity to conventional radiotherapy and chemotherapy, immunotherapy and targeted therapy were used as first-line treatment for patients with ccRCC metastases, but the prognosis remained poor [4]. Therefore, in order to provide better treatment for patients, it was urgent to obtain a deeper understanding of the biological mechanisms of ccRCC.

Recently, immune checkpoint inhibitors (ICIs), including antibodies targeting programmed death-1 (PD-1) receptor, its ligand (PD-L1), and cytotoxic T lymphocyte associated protein 4 (CTLA-4), have become the main means of cancer treatment [5, 6]. Immunotherapy has been proven to be an effective and important new strategy for the management of patients with ccRCC [7–9]. However, only a few patients with ccRCC benefited from immunotherapy. This phenomenon may be attributed to the complex and different immune microenvironment among individuals with this type of tumor [10, 11]. In reality, it was difficult to predict the response of patients with ccRCC to immunotherapy. The predictive value of tumor PD-L1 expression and tumor mutational burden (TMB) as auxiliary diagnostic biomarkers for other tumor types remained doubtful in cases of ccRCC [12], since many studies have shown that this did not correlate with the response of patients with ccRCC to immunotherapy [13]. Consequently, the identification of reliable biomarkers for immunotherapy response was urgent, to promote the improvement of clinical efficacy for these therapies [14].

The purpose of this study was to describe the immune characteristics of ccRCC and define each population, to propose new treatment options. Based on the clustering of immune related genes, we defined three immune subtypes. Each immune subtype corresponded to diverse clinical and molecular characteristics. Our findings revealed the complex tumor immune microenvironment in each patient with ccRCC and screened antigens for subtype classification, which provided a theoretical basis for the selection of appropriate patients for immunotherapy.

## RESULTS

### Identification of immune characteristics of ccRCC and para-cancerous renal tissues

We first used single sample gene set enrichment analysis (ssGSEA) to identify differences in immune cell infiltration between normal and ccRCC tissues. As shown in Supplementary Figure 1A, all immune cells had diverse degrees of differential expression between normal and ccRCC tissues. Moreover, by uploading the normalized ccRCC expression matrix to the CIBERSORT website, we obtained the relative proportions of 22 types of immune infiltrating cells in ccRCC. Then, the Wilcoxon test was applied to ascertain the differences in infiltrating immune cells, with P <0.05 as the threshold. The result indicated that there were significant differences in the expression of infiltrating immune cells between ccRCC and normal tissues (Supplementary Figure 1B). Previous studies have shown that immune checkpoints (ICPs) and immunogenic cell death (ICD) modulators play important roles in tumor immunity. Therefore, in order to explore the immune characteristics of normal and ccRCC patients, the expression of ICPs and ICD modulators was evaluated. Similar to the previous results, almost all ICPs and ICD modulators were differently expressed in the two groups (Supplementary Figure 1C, 1D). These results confirmed that there were significant differences in the immune environment between ccRCC and normal tissue. In order to further verify our results, the algorithm of estimation of stromal and immune cells in malignant tumors using expression data (ESTIMATE) was used to calculate the immune microenvironment scores, including the immune score, stromal score, and estimate score. It was obvious that the immune score, stromal score, and estimate score were significantly more expressed in ccRCC tissues, which indicated that the immune microenvironment was more complex in cancer than normal tissue samples (Supplementary Figure 1E).

### Identification of immune subtypes of ccRCC

**Figure 1:**
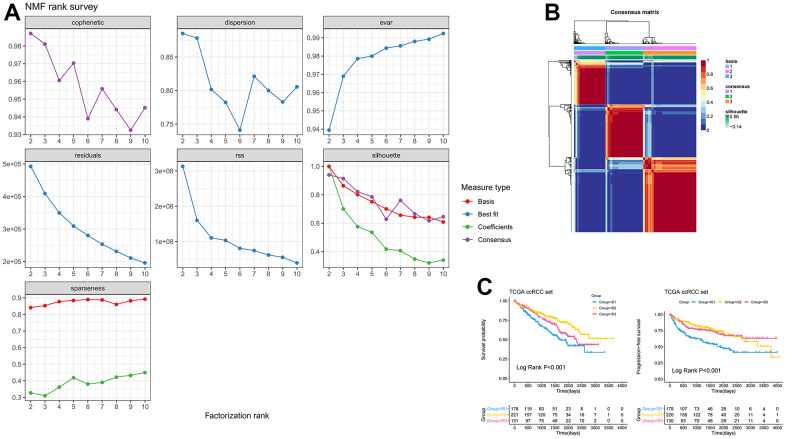
**Identification of three distinct immune-related molecular subtypes of ccRCC in the TCGA-ccRCC dataset by NMF.** (**A**) Cumulative distribution function curve and (**B**) delta area of immune-related genes in the ICGC cohort. (**C**) Kaplan-Meier curves showing OS and PFS of ccRCC immune subtypes in the TCGA cohort.

Given that there were significant differences in the immune environment between ccRCC and normal tissues, 186 differentially expressed genes were screened from 1307 immune-related genes with a criterion of |LogFC| >2, p <0.05, and then these genes were used to construct immune subtypes. Non-Negative Matrix Factorization (NMF) and Principal Component Analysis (PCA) are both decomposition algorithms, used to find optimal linear projections of data onto lower dimensional space without losing much information about the structure within the data [15]. Another well-known manifold learning algorithm is t-Distributed Stochastic Neighbor Embedding (t-SNE), which allows visualization of data in low dimensional space without losing too much information [16]. Compared to other algorithms, NMF provides “meta features” (or “meta genes”) that represent the main characteristics of the whole data, enabling more effective characteristic clustering [17–19]. PCA and t-SNE are generally used for dimensionality reduction and visualization. NMF can also be used for dimensionality reduction, but it is more suitable for clustering [20]. Eventually, three immune subtypes (e.g., IS1, IS2, IS3) were identified based on the NMF algorithm (Figure 1A, 1B). Supplementary Table 1 showed the subtypes for the samples. The overall survival (OS) results showed that patients in the IS1 subgroup had the worst survival, while patients in the IS2 subgroup had the best survival. This result was consistent with progression free survival (PFS) (Figure 1C), which confirmed that the molecular subtypes not only play well-differentiated roles in patients with ccRCC, but may also serve as prognostic biomarkers. Additionally, we downloaded the E-MTAB-1980 data set from ArrayExpress to further verify the stability and reliability of the immune subtypes of ccRCC. Three immune subtypes were identified by the NMF algorithm (Supplementary Figure 2A). The Kaplan-Meier (KM) survival analysis indicated that there were significant differences in survival among the three immune subtypes (Supplementary Figure 2B).

### Identification of the association between mutation characteristics of immune subtypes with other biological biomarkers

**Figure 2:**
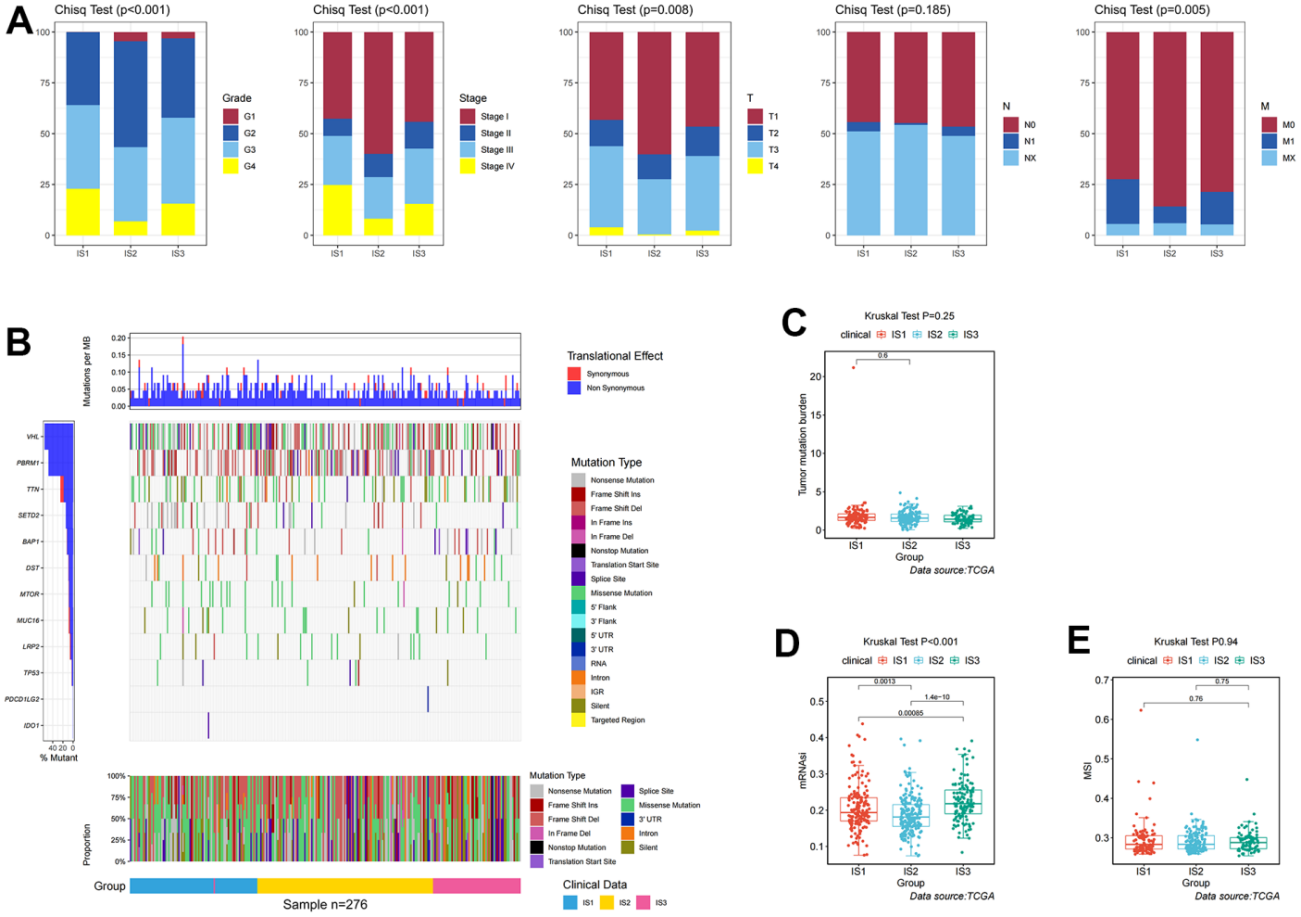
**Identification of mutations, immune, and clinicopathological differences among three distinct ccRCC immune-related molecular subtypes.** (**A**) Distribution ratio for grades, stages, T stages, N stages, and M stages across IS1–IS3 in the TCGA cohort. (**B**) Distribution landscape of mutation and CNV among IS1–IS3 in the TCGA cohort. (**C**) Distribution landscape of tumor mutation burden among IS1–IS3 in the TCGA cohort. (**D**) Distribution landscape of mRNAsi status among IS1–IS3 in the TCGA cohort. (**E**) Distribution landscape of MSI status among IS1–IS3 in the TCGA cohort.

In addition, differences in clinicopathological characteristics between immune subtypes were further analyzed. In IS1 patients, the proportion of advanced clinicopathological characteristics was significantly higher than that of IS2 and IS3 patients, but there was no significant difference in the degree of lymph node involvement among the three groups (Figure 2A). The distribution of the top ten mutants in each population was plotted (Figure 2B). TMB was an effective marker for predicting the response to immunotherapy, and was applied to identify the characteristics of each immune subtype. As shown in Figure 2C, the IS1 group had a higher TMB, followed by the IS2, and the IS3 had a lower TMB, which suggested that IS3 patients were more likely to respond to immune checkpoint inhibitors. However, there was no significant difference in TMB among these three immune subtypes (p =0.28, Figure 2C). After appraising the mRNA expression-based stemness index (mRNAsi), which referred to the similarity between tumor cells and cancer stem cells (CSCs), the results suggested that IS3 had the highest mRNAsi, followed by IS1 and IS2 (p <0.001, Figure 2D). Microsatellite instability (MSI) was characterized by a high frequency of insertions/deletions due to unrepaired DNA polymerase slippage in microsatellite sequences, and may be applied to predict immunotherapy response. The results suggested that IS3 patients had the highest MSI (p =0.94), indicating IS3 patients display a better immunotherapeutic response (Figure 2E).

### Identification of the immune characteristics of each immune subtype

**Figure 3:**
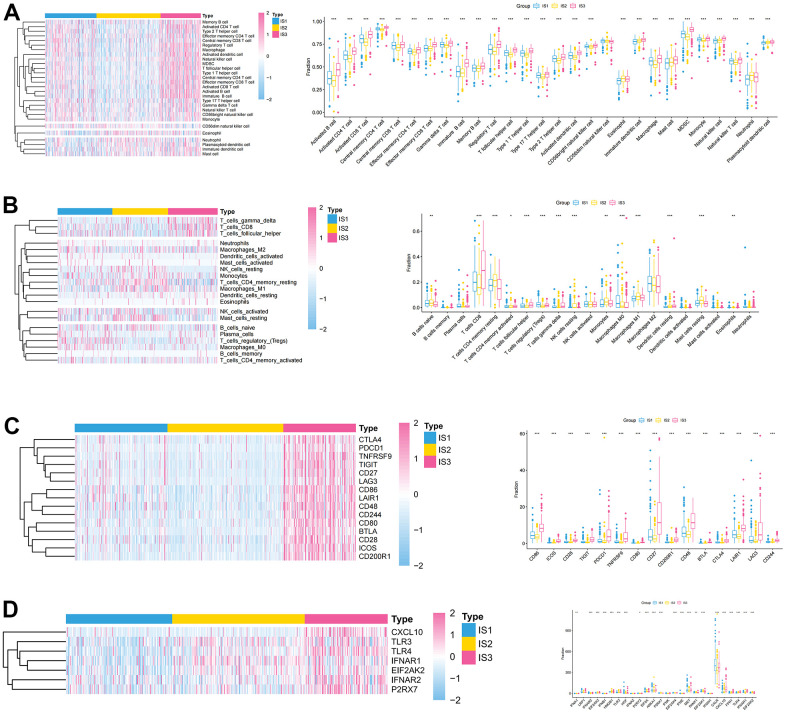
**Association between immune subtypes, immune cells, and immune checkpoints.** (**A**, **B**) Differential expression of immune cells among the ccRCC immune subtypes (**A**) ssGSEA; (**B**) CIBERSORT. (**C**) Differential expression of immune checkpoints among the ccRCC immune subtypes in TCGA cohorts. (**D**) Differential expression of ICD modulators among the ccRCC immune subtypes in TCGA cohorts.

To explore the immune characteristics of the three immune subtypes, we used the CIBERSORT and ssGSEA algorithms to analyze the distribution and expression differences of immune cells. After assessing immune cell infiltration in ccRCC samples by ssGSEA, most immune cells were significantly over-expressed in the IS3 group, while in the IS2 group a large number of these immune cells were significantly under-expressed (Figure 3A). Furthermore, we used CIBERSORT to assess the expression of 22 immune infiltrating cells in immune subtypes. Individual immune infiltrating cells were substantially varied in these three subtypes. The expression of regulatory T cells (Tregs) and M0 macrophages was significantly increased in IS1, while the expression of B cells, NK cells, and mast cells was higher in IS2 (Figure 3B). The association between ICPs, ICD modulators, and these three immune subtypes was also explored. Most of the immune checkpoints were highly expressed in IS3, such as PDCD1 and CTLA4 (Figure 3C). Concomitantly, there were also significant differences in the expression of ICD regulators between the three immune subtypes, with higher expression in IS3 (Figure 3D). These results suggested that IS3 was a “hot” immune phenotype, IS2 was a “cold” immune phenotype, and IS1 was an “intermediate” immune phenotype. Therefore, this immune subtype may be used to distinguish the immune status of patients with ccRCC.

### Identification of immunotherapy regimens for each subtype

**Figure 4:**
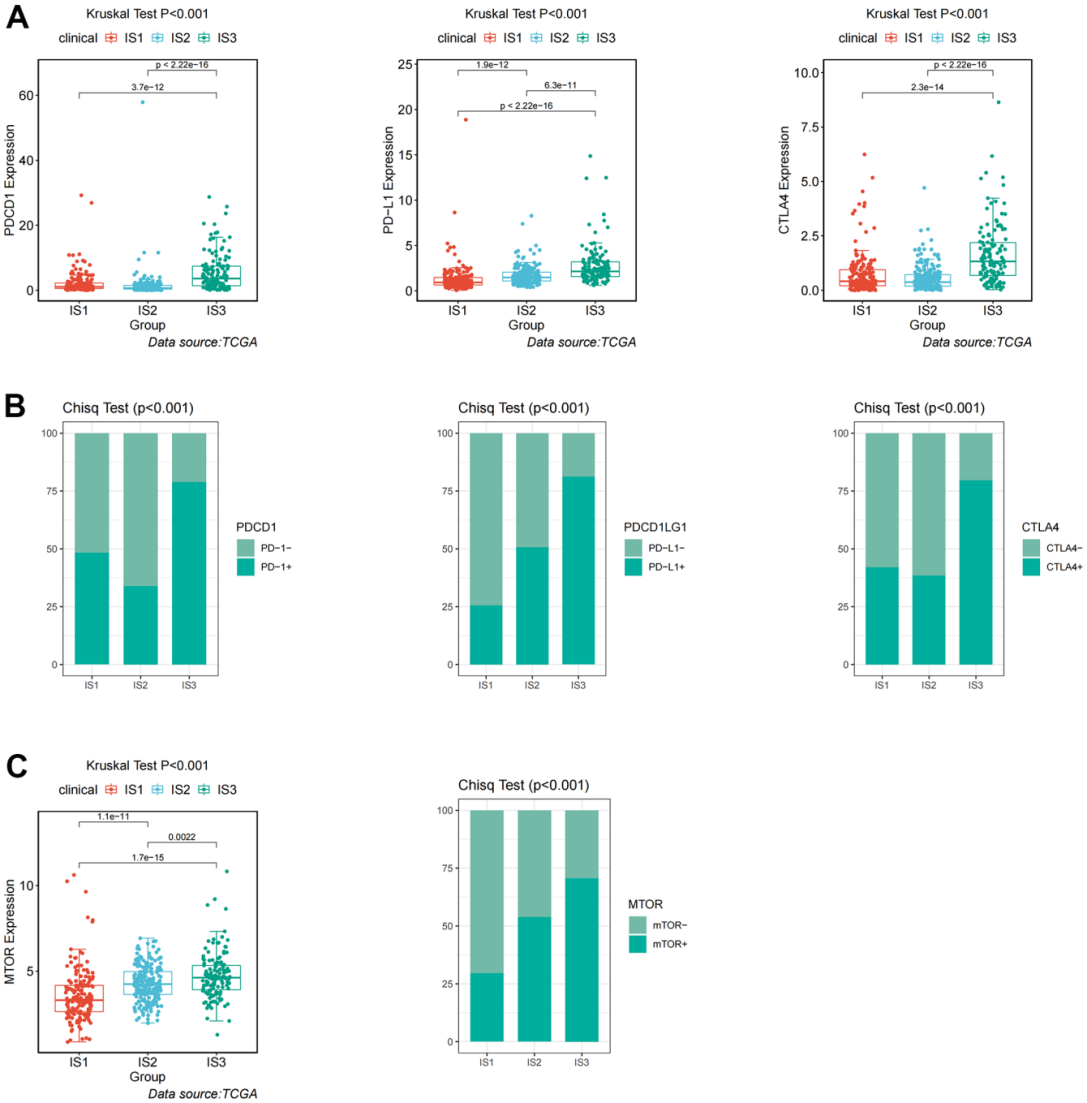
**Identification of differential expression of immune checkpoints and mTOR among immune-related subtypes.** (**A**) Differential expression of PDCD1, PD-L1, and CTLA4 among the ccRCC immune subtypes in TCGA cohorts. (**B**) Differential proportion of PDCD1^+^, PD-L1^+^, and CTLA4^+^ among the ccRCC immune subtypes in TCGA cohorts. (**C**) Differential expression and proportion of mTOR^+^ among the ccRCC immune subtypes in TCGA cohorts.

**Figure 5:**
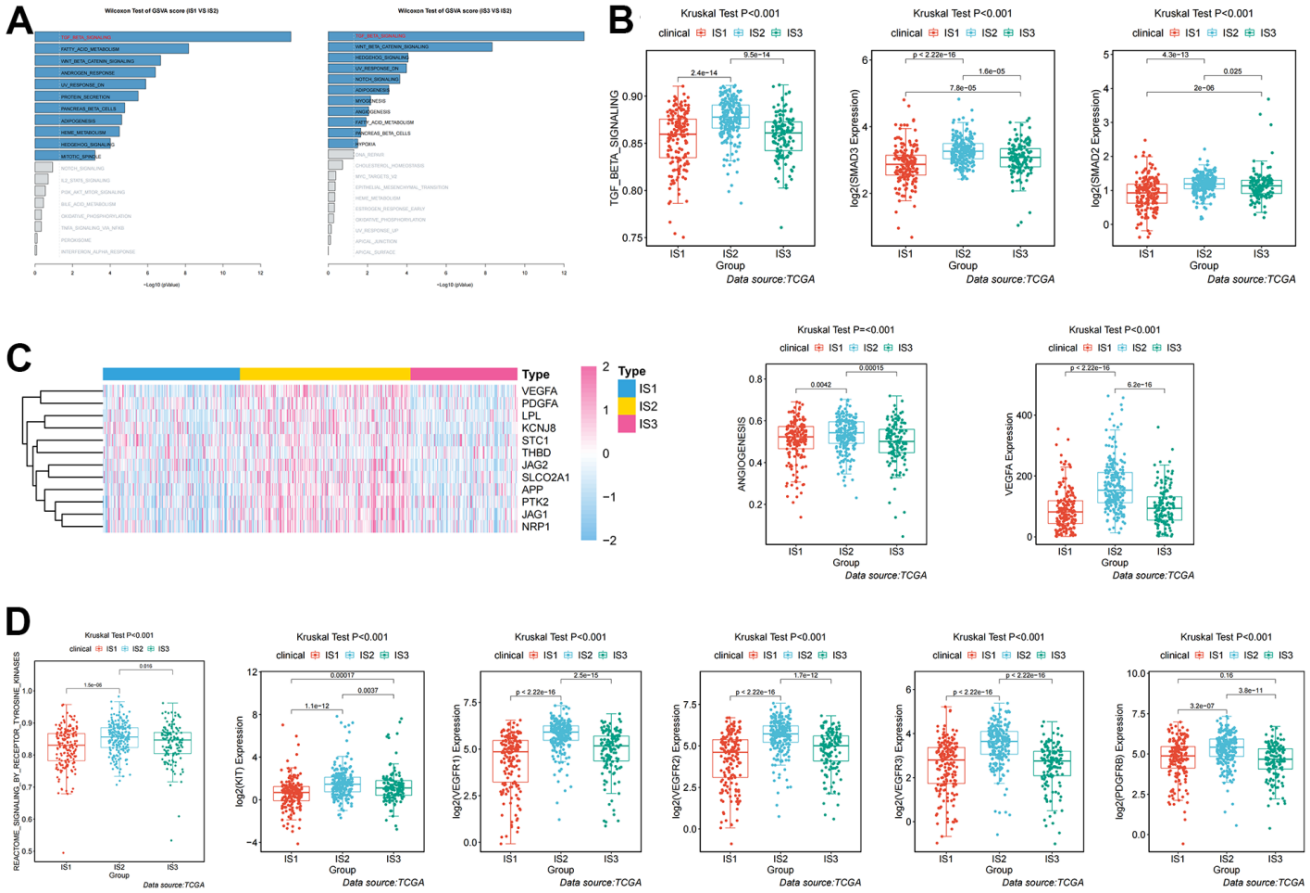
**Analysis and definition of biological and immunological characteristics of IS2.** (**A**) Differential enrichment analysis of signaling pathways in IS2 compared with IS1 and IS3 in TCGA cohorts. (**B**) Differential expression of the TGF-β pathway and key regulators (SMAD2 and SMAD3) among immune-related subtypes. (**C**) Differential expression of the ANGIOGENESIS pathway and major regulatory genes among immune-related subtypes. (**D**) Differential expression of the REACTOME SIGNALING BY RECEPTOR TYROSINE KINASES pathway and major regulatory genes among immune-related subtypes.

**Figure 6:**
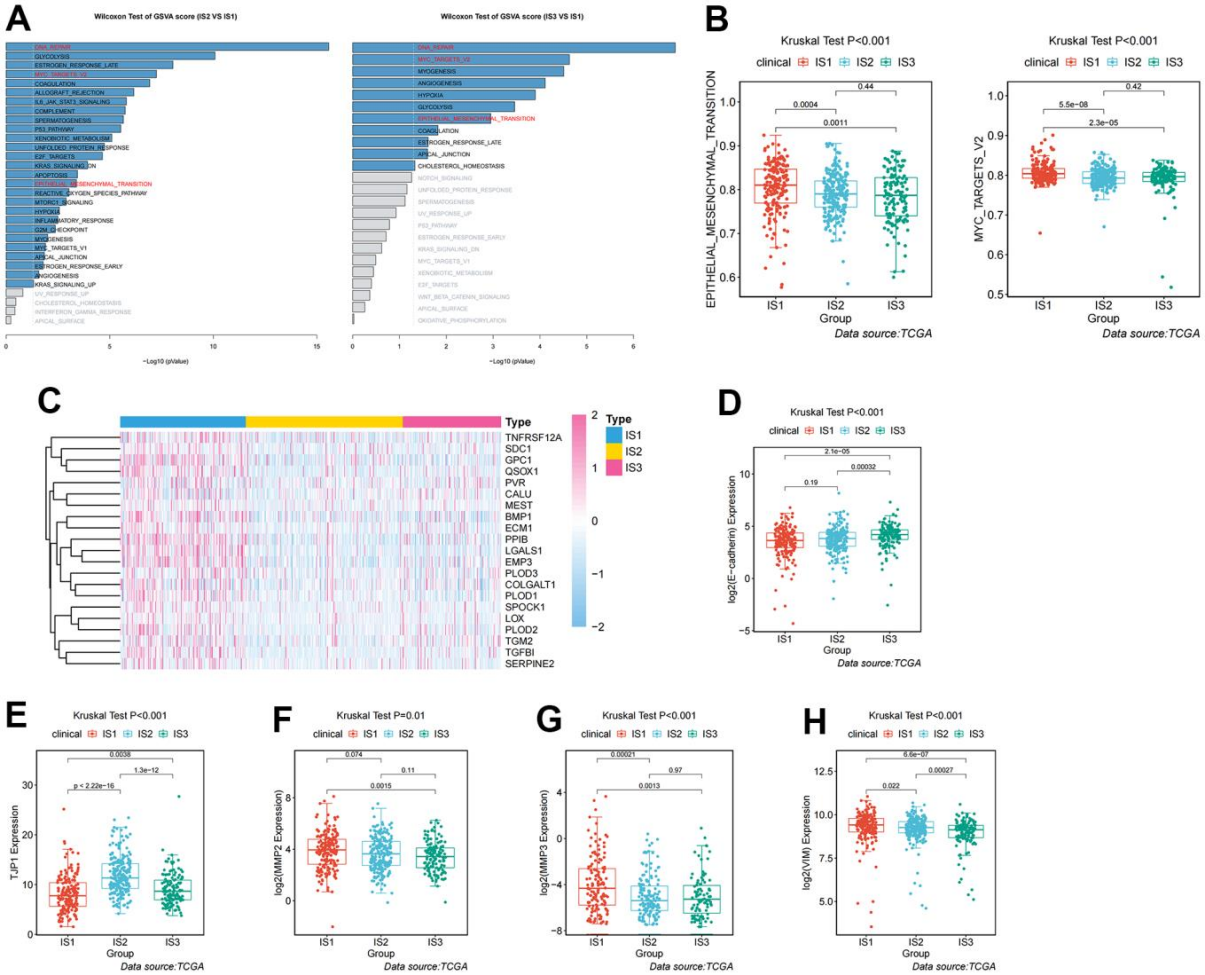
**Analysis and definition of biological and immunological characteristics for IS1.** (**A**) Differential enrichment analysis of signaling pathways in IS1 compared with IS2 and IS3 in TCGA cohorts. (**B**) Differential enrichment analysis of EMT and Myc signaling pathways among the ccRCC immune subtypes in TCGA cohorts. (**C**) Heatmap showing the distribution of key regulators of the EMT signaling pathway among the three immune subtypes. (**D**–**H**) Differential expression of molecules in the EMT signaling pathway among the three immune subtypes in TCGA cohorts.

Given the high level of immune complexity of the three immune subtypes, we calculated differences in expression of the most common targets (PD-1, PD-L1, and CTLA4) across immune subtypes, in order to identify potential immunotherapeutic regimens for patients. In IS3, the expression of PD-1, PD-L1, and CTLA4 were higher than in IS1 and IS2 (Figure 4A). Moreover, the ccRCC samples were divided into positive and negative groups, according to the median value of the most common target expression. The ratio plot revealed that the number of highly expressed targets (PD-1^+^, PD-L1^+^, and CTLA4^+^) had the highest proportion in IS3 (Figure 4B). In addition, mammalian target of rapamycin (mTOR), a multi-tumor therapeutic target, was also evaluated. We discovered the abnormally high expression of mTOR in IS3, and the proportion of highly expressed mTOR was the highest in IS3 (Figure 4C). Due to the significantly high expression of immune checkpoints and mTOR, patients in the IS3 group were considered suitable for the combination of immune checkpoints (PD-1, PD-L1, and CTLA4) and mTOR inhibitors, which has been clinically proven to be one of the best immunotherapy regimens available. We further analyzed the differences in biological pathways among the three subtypes, and found that the TGF_BETA_SIGNALING pathway was significantly enriched in the IS2 subtype (Figure 5A). Additionally, the TGF_BETA_SIGNALING pathway and key regulatory genes (SMAD3 and SMAD2) were also observably over-expressed in the IS2 subtype compared to IS1 and IS3 (Figure 5B). We further analyzed expression differences for the ANGIOGENESIS pathway and major regulatory genes among immune subtypes. The results showed that the expression of the ANGIOGENESIS pathway and major regulatory genes were the highest in IS2 (Figure 5C). The same trend was observed in the REACTOME SIGNALING BY RECEPTOR TYROSINE KINASES pathway. Then, we inspected the expression of the tyrosine kinase inhibitor (TKI) pathway targets in the IS2 subtype. It was obvious that the expressions of VEGFA, VEGFR1, VEGFR2, VEGFR3, PDGFRB, and KIT were significantly over-expressed in the IS2 subtype (Figure 5D). Therefore, patients with IS2 ccRCC may be treated with a combination of TKI targeted therapy and antiangiogenic therapy. Given that cases in the IS1 subtype have a higher proportion of patients with a metastatic propensity, we therefore compared the IS1 subtype with the IS2 and IS3 subtypes. The results showed that the DNA_REPAIR, MYC_TARGETS_V2 (MYC), and epithelial-to-mesenchymal transition (EMT) pathways were significantly enriched in IS1 (Figure 6A). Given that IS1 had the highest proportion of metastases, we focused on pathways significantly associated with tumor metastasis (MYC and EMT). Then, we further analyzed the expression differences for EMT and MYC among immune subtypes, which showed that EMT and MYC were the highest in IS1 (Figure 6B). In order to develop a treatment plan for patients with IS1 ccRCC, we analyzed the receptors and drug targets of these signaling pathways, and found that E-cadherin, MMP2, MMP3, and VIM were significantly highly expressed in the IS1 subtype, while TJP1 (ZO-1) had significantly lower expression (Figure 6D–6H). These results revealed that the IS1 population may respond to a combination of EMT inhibitors with c-Myc inhibitors. These subtype characteristics may provide the basis for future clinical treatment.

### Identification of antigens as molecular predictor of immune subtypes

**Figure 7:**
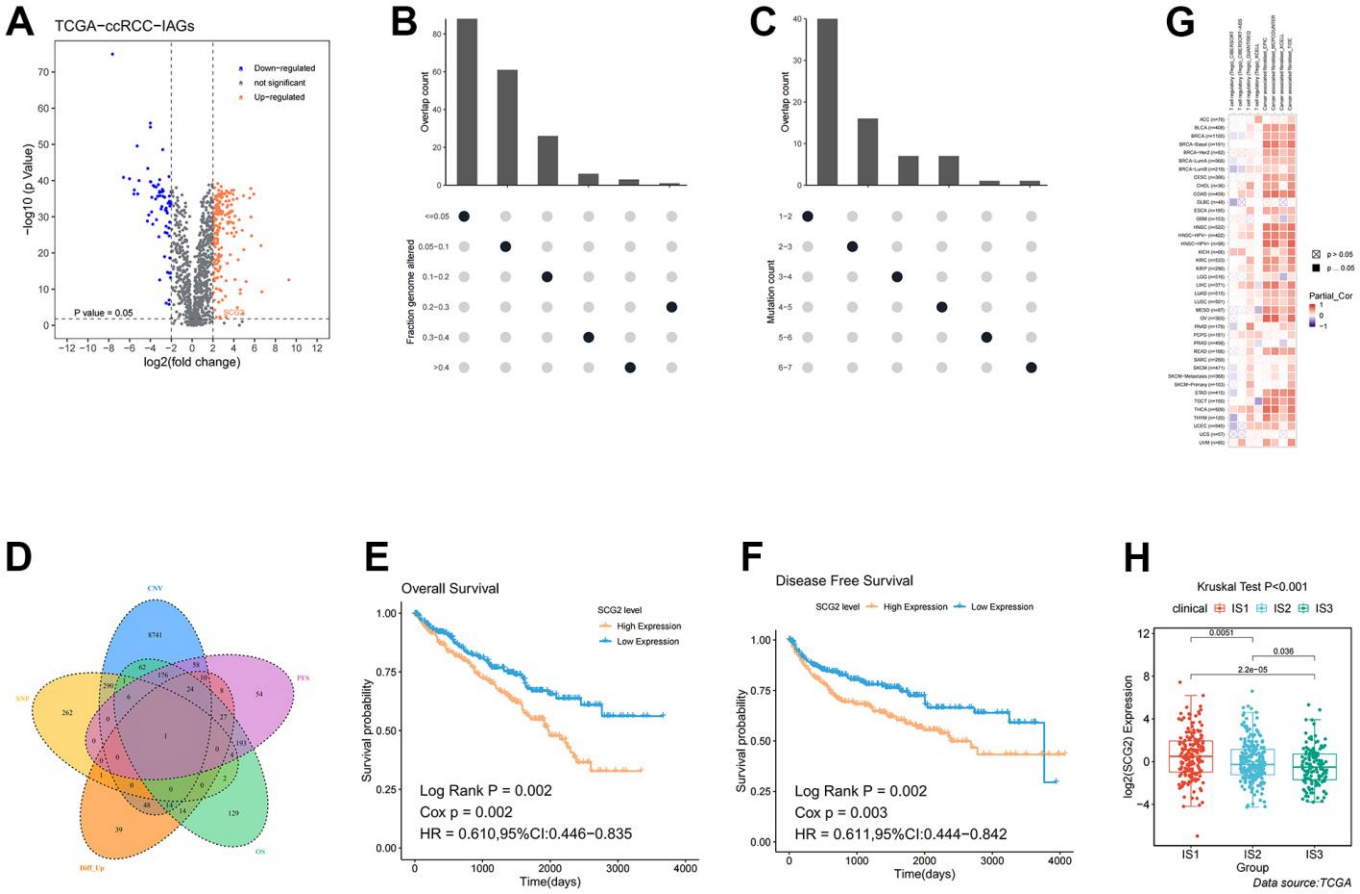
**Identification of potential tumor antigens in ccRCC.** (**A**) Identification of potential tumor-associated antigens in ccRCC through differential expression analysis. Chromosomal distribution of up- and down-regulated genes in ccRCC, as indicated. (**B**, **C**) Identification of potential tumor-associated antigens in ccRCC through fraction of the genome altered and mutation analysis. (**D**) Combined analysis of multiple spectra to identify specific antigens. (**E**, **F**) The association of SCG2 with OS and PFS. (**G**) The correlation between SCG2 and immune cells (Tregs and CAFs) in pan-cancer. (**H**) Differential expression of SCG2 among the ccRCC immune subtypes in TCGA cohorts. Tregs: regulatory T cell; CAFs: cancer associated fibroblast.

Although we have identified the immune characteristics and the treatment regimens for each subtype, we also need to identify a tumor antigen that can predict patient prognosis and differentiate the three immune subtypes. The volcano plot presented the distribution of differentially expressed immune-associated genes (IAGs) (Figure 7A). Histograms showed the number of fraction genome altered and mutations in ccRCC samples (Figure 7B, 7C). Among these differentially increased IAGs, combined with screening conditions such as fraction genome altered >0.1, and total mutation number >5, we finally identified the most likely potential tumor antigen: SCG2 (Figure 7D). Thus, the KM curve revealed that high expression of SCG2 was associated with poor OS and disease-free survival (DFS) prognoses (Figure 7E, 7F). Furthermore, the association of Tregs and cancer-associated fibroblasts (CAFs) with SCG2 in various tumors was assessed by multiple algorithms. Interestingly, SCG2 was significantly positively associated with Tregs and CAFs (Figure 7G). We also investigated the expression of SCG2 in three immune subtypes, and we found that SCG2 may distinguish the subtypes well, which may help define new patient subtypes in the future (Figure 7H).

### Identification of the immunological and clinical characteristics of SCG2

**Figure 8:**
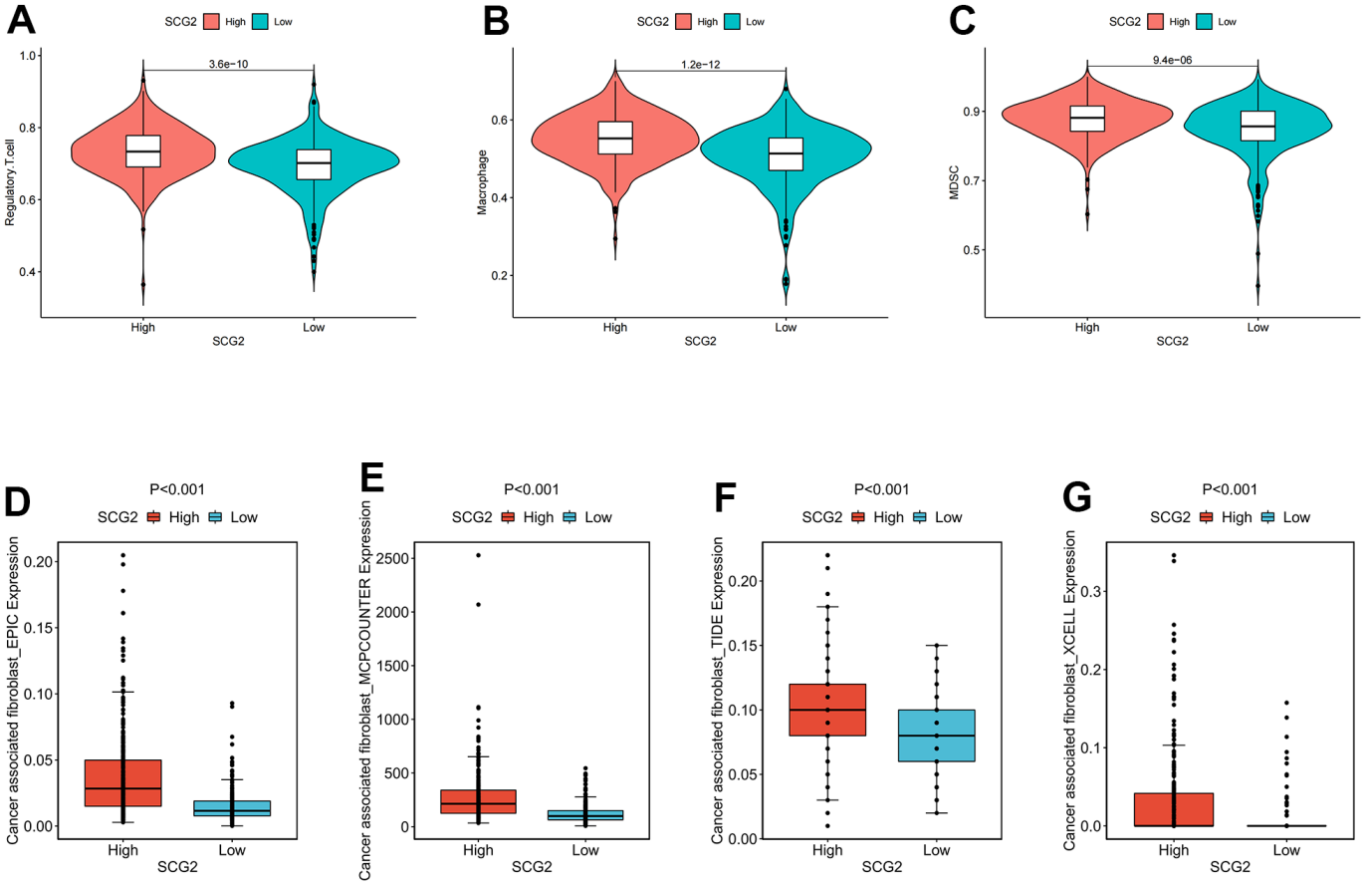
**Identification of immunological characteristics of SCG2.** (**A**–**C**) Correlation between SCG2 and immunosuppressive cells. (**A**) Tregs, (**B**) macrophage, (**C**) MDSC. (**D**–**G**) Correlation between SCG2 and CAFs in various algorithms. (**D**) EPIC, (**E**) MCPCOUNTER, (**F**) TIDE, (**G**) XCELL.

To further investigate the immune signature of SCG2, we first scored immune cells in tumor tissues from 539 patients with ccRCC. The results indicated that SCG2 was significantly positively correlated with immune cells, especially MDSC, macrophages, and Tregs (Figure 8A–8C). Then, CIBERSORT, EPIC, TIDE, and XCELL algorithms were employed to analyze the correlation between SCG2 and CAFs. The results revealed that SCG2 was positively correlated with CAFs (Figure 8D–8G). Then, to further validate the key events leading to tumor development, we analyzed the correlation between SCG2 expression and clinicopathological variables. In the Cancer Genome Atlas (TCGA) cohort, a high expression level of SCG2 was significantly associated with more severe clinical predictors, including histological grade (p <0.001), T stage (p <0.001), and M stage (p <0.05) (Supplementary Figure 2C–2F). The results from the same calculations in the GSE17895, GSE53537, GSE73731, and GSE40435 datasets revealed that SCG2 was significantly over-expressed in patients with ccRCC and the higher the histological grade and pathological stage, the higher the expression of SCG2 (Supplementary Figure 2G–2K).

## DISCUSSION

ccRCC is a heterogeneous disease with different ethnic characteristics, which originated from renal tubule epithelial cells [21]. It is estimated that ccRCC accounted for a considerable part of cancer related mortality [22]. Now more and more evidence shows that ccRCC has a unique immune microenvironment compared with other solid tumors. The resected ccRCC was usually extensively infiltrated by CD8^+^ T cells, which indicated the immune recognition of the tumor [23]. Therefore, the unique characteristics of ccRCC made it an attractive disease for the treatment with ICI [24]. The absence of VHL in most ccRCC led to the increase of VEGF, which promotes immunosuppression as well as angiogenesis [25]. An important area underway was to adapt ICI to these tumor-specific immune characteristics [26, 27]. Therefore, we investigated the immune microenvironment of ccRCC, and stratified patients, in order to identify appropriate groups for immunotherapy.

Three immune subtypes were clustered through the NMF algorithm, according to the immune gene expression profile, to select the appropriate population for immunotherapy. The three immune subtypes manifested different clinical, cellular, and molecular characteristics. In a previous study, two hypoxia-related molecular subtypes in ccRCC were constructed with the NMF algorithm, which presented different biological and immune signatures [28]. In addition, Zhang et al. analyzed the expression of pyroptosis-related genes in ccRCC tissues [29]. Based on the pyroptosis components, they divided ccRCC patients into four pyroptosis subtypes with distinct clinical, molecular, and pathway characteristics. The subtype can be used as a predictor of immunotherapy response. In a separate study, Wang et al. screened 49 adipose-related genes, which were differentially expressed between normal and ccRCC tissues [30]. Based on differentially expressed adipose-related genes (ARGs), patients with ccRCC were divided into two adipose subtypes, with distinct clinical, molecular, and pathway characteristics. ARG may aid in the development of novel biomarkers and immunotherapies for ccRCC patients. Unlike these studies, our study used immune-related genes. The subtypes established in this way are more closely related to immune characteristics, which is more suitable for finding potential populations for whom immunotherapy is applicable.

Since tumor immune status is the determinant of immunotherapy response, we further characterized the immune cell components in different subtypes [31, 32]. Compared with IS2 and IS1, IS3 had abundant immune cell infiltration and complex immune microenvironment. These results indicated that IS3 was a “hot” immune phenotype, IS2 was a “cold” immune phenotype, and IS1 was an “intermediate” immune phenotype. The molecular characteristics of these subtypes were consistent with the immune characteristics, indicating that patients with different immune subtypes have different treatment responses. Specifically, the IS3 subtype was associated with higher immune cell infiltration, suggesting more abundant immune components. Immune checkpoints are a class of immunosuppressive molecules that regulate immune responses to avoid the destruction of normal tissues [33, 34]. During tumorigenesis, immune checkpoints are important biomarkers of the immune cell phenotype in the immune microenvironment, which can induce immune cells to develop immune tolerance [35, 36]. Our analysis showed that the IS3 subtype has a high expression of immune checkpoints (PD-1, PD-L1, and CTLA-4), which may afford it a better response to immune checkpoint inhibitors. Furthermore, we found that the mTOR signaling pathway was significantly enriched in the IS3 subtype, suggesting that immune checkpoint inhibitors combined with mTOR inhibitors may be used to treat patients in the IS3 subtype.

Among the three subtypes, IS1 had the worst prognosis, which may be related to the highest proportion of patients with M1 stage in IS1. Studies have shown that a higher clinical stage may lead to poorer tumor prognosis [37, 38]. In addition, enrichment results showed that the EMT signaling pathway was abnormally enriched in IS1. EMT is a cellular process in which epithelial cells acquire the phenotype and behavior of stroma, after downregulating epithelial characteristics [39–41]. During EMT, tumor cells showed fibroblast-like morphology and cell structure, as well as increased migration and invasion abilities. Furthermore, we found that c-Myc was highly expressed in IS1. The c-Myc family of oncoproteins were the main driving force of human tumorigenesis, which is promoted by regulation of transcription mechanisms [42]. Previous studies have shown that c-Myc phosphorylation mediated by PIM1 activates the expression of ZEB1, ZEB2, Snail 1, Snail 2, and Twist transcription factors to promote EMT in RCC [43]. Intrinsically, these signaling pathways may be the underlying reasons for the high metastatic propensity and poor prognosis of IS1.

To guide the differentiation and treatment of the three subtypes, we screened tumor antigens. The results of screening showed that SCG2 was associated with tumorigenesis and progression at multiple levels, and had good prognostic significance. SCG2 was a member of the chromogranin / secretory granulin family of neuroendocrine proteins, which was involved in packaging peptide hormones and neuropeptides into secretory vesicles. Simultaneously, SCG2 has been proven to be abnormal regulation in the occurrence and development of a variety of malignant tumors [44–48]. According to our analysis, SCG2 was differentially expressed among the three subtypes. We proposed a scientific assumption: in clinical transformation, patients may be classified and defined according to the expression of SCG2, and then be treated according to the treatment schemes of the three subtypes mentioned above.

However, there were still some shortcomings in this study. On the one hand, TIMER 2.0 was utilized to evaluate the correlation between gene expression and immune/stromal cell infiltration. The number of tumor cases in TCGA and TIMER2.0 databases may be inconsistent. Fortunately, there are only a few inconsistent cases, and the deviation can be ignored to some extent. In addition, this immune correlation was also verified by TCGA data. On the other hand, the p-value was used for screening differentially expressed genes rather than adjusted p-values. This may include false positive genes, but it also reduces the elimination of true positive genes.

The current study systematically revealed the different immune landscapes in ccRCC and adjacent tissues through integrated bioinformatics methods. Furthermore, a novel immune subtype was established, and the biological characteristics of these subtypes were determined. In addition, a potential antigen (SCG2) related to immune subtypes was identified, which showed significant immune correlation. These findings provided new insights into the immunological mechanism of ccRCC biology and the refined disease management of patients.

## MATERIALS AND METHODS

### Acquisition of data

Transcriptome data and somatic mutation information of patients with ccRCC were collected from the TCGA database (https://portal.gdc.cancer.gov/) as the training dataset, including 72 normal samples and 539 tumor samples. Meanwhile, the corresponding clinical data were recorded in Table 1. The gene expression data and clinical information retrieved from the International Cancer Genome Consortium (ICGC) database were utilized as the external validation dataset. A total of 2498 immune genes were downloaded from the ImmPort database, of which there were only 1307 immune genes in the TCGA expression profile. The “Limma” package was used to correct the data and process the repeated gene expression data. The Wilcoxon test was applied to identify differentially expressed immune genes. Meanwhile, |LogFC| >2 and p <0.05 were used as the criteria for screening differentially expressed immune genes.

**Table 1:** Clinical characteristics of included patients in the study.

**Variables**	**Total (n=525)**
Age (year)	
<65	347(66.1%)
≥65	178(33.9%)
Gender	
FEMALE	182(34.67%)
MALE	343(65.33%)
Stage	
I	261(49.71%)
II	56(10.67%)
III	123(23.43%)
IV	82(15.62%)
unknow	3(0.57%)
T stage	
T1	267(50.86%)
T2	68(12.95%)
T3	179(34.1%)
T4	11(2.1%)
N stage	
N0	237(45.14%)
N1	16(3.05%)
NX	272(51.81%)
M stage	
M0	417(79.43%)
M1	78(14.86%)
MX	28(5.33%)
unknow	2(0.38%)
Grade	
G1	13(2.48%)
G2	226(43.05%)
G3	204(38.86%)
G4	74(14.1%)
GX	5(0.95%)
unknow	3(0.57%)

### Establishment of immune subtypes

Based on the expression of the screened immune genes, we employed the NMF algorithm to cluster ccRCC samples. The “Brunet” method was applied to select the best number for clustering, and the number of iterations was 30. The point with the first greatest variation of cophenetic value was considered the best number of immune subtypes. In addition, OS and PFS were used to evaluate the reliability of the clustering results. The KM method was employed to analyze differences in survival between different subtypes. All statistical p-values were two-sided, with p <0.05 as statistically critical.

### Identification of immune characteristics of subtypes

The hallmark gene sets containing 50 different biological pathways including APOPTOSIS, MTORC1_SIGNALING, etc. were downloaded from the MSigDB, and ssGSEA was applied to score the gene lists in these pathways. CIBERSORT was a deconvolution algorithm which used 547 tag gene expression values to characterize the composition of immune cells in tissues. To assess the association between these immune subtypes, the CIBERSORT algorithm was applied to estimate the relative proportion of 22 immune infiltrating cells in patients with ccRCC. We uploaded the corrected transcriptome data to the CIBERSORT website (http://cibersort.stanford.edu/) and set the algorithm to 1000 rows. P <0.05 was used as the criteria. The immune score and stromal score, which contained all stromal cells including CAFs, endothelial cells (ECs), mesenchymal stem cells (MCSs), and pericytes, were calculated by the ESTIMATE algorithm.

### TIMER analysis

TIMER was an open resource for evaluating the proportion of various immune infiltrating cells across diverse cancer types. In this study, TIMER2.0 was employed to visualize the correlation between CAF infiltration, Tregs, and the identified potent antigens. The correlation between diverse immune infiltrating cells and the identified potent antigens was calculated by Spearman correlation analysis. P <0.05 was used as the criteria, and the correlation value varied from -1 to 1, so that the larger the absolute value, the more relevant.

## Supplementary Material

Supplementary Figures

Supplementary Table 1
