# Supplementary material for: Identification of immune subtypes to guide immunotherapy and targeted therapy in clear cell renal cell carcinoma
Source: Aging (Albany NY). 2022 Sep 1;14(17):6917–35. doi: 10.18632/aging.204252 (PMC9512512; doi:10.18632/aging.204252)
Supplement: Supplementary Figures [file aging-14-204252-s001.pdf]

## SUPPLEMENTARY FIGURES

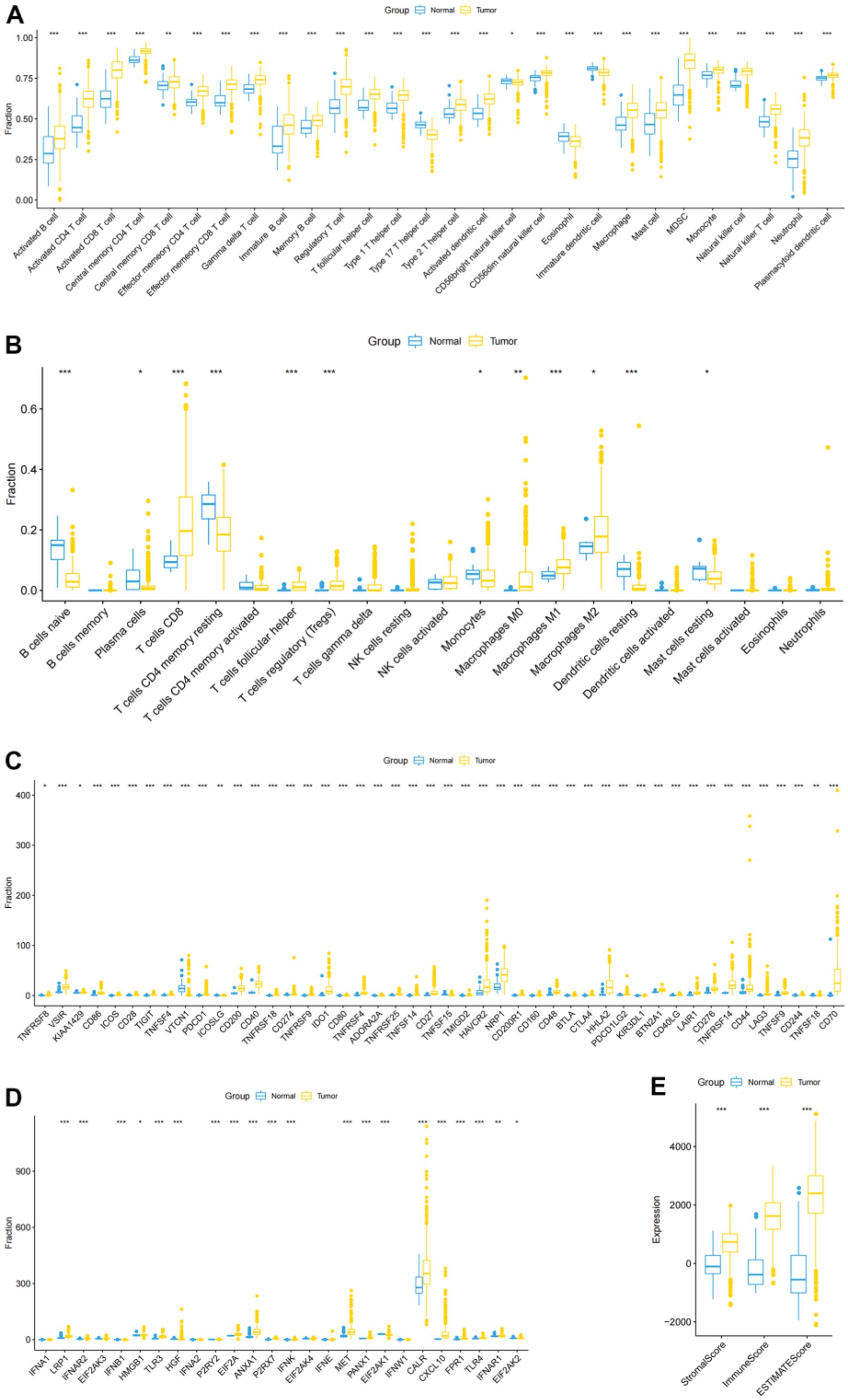


**Supplementary Figure 1. The atlas and landscape of immune infiltration in a ccRCC cohort.** (**A**, **B**) Differential expression of immune-infiltrating cells in ccRCC and para-cancerous tissues (**A**) ssGSEA; (**B**) CIBERSORT. (**C**) Differential expression of immune checkpoints in ccRCC and para-cancerous tissues. (**D**) Differential expression of ICD modulators in ccRCC and para-cancerous tissues. (**E**) Characteristics of tumor microenvironment in ccRCC and para-cancerous renal tissue.

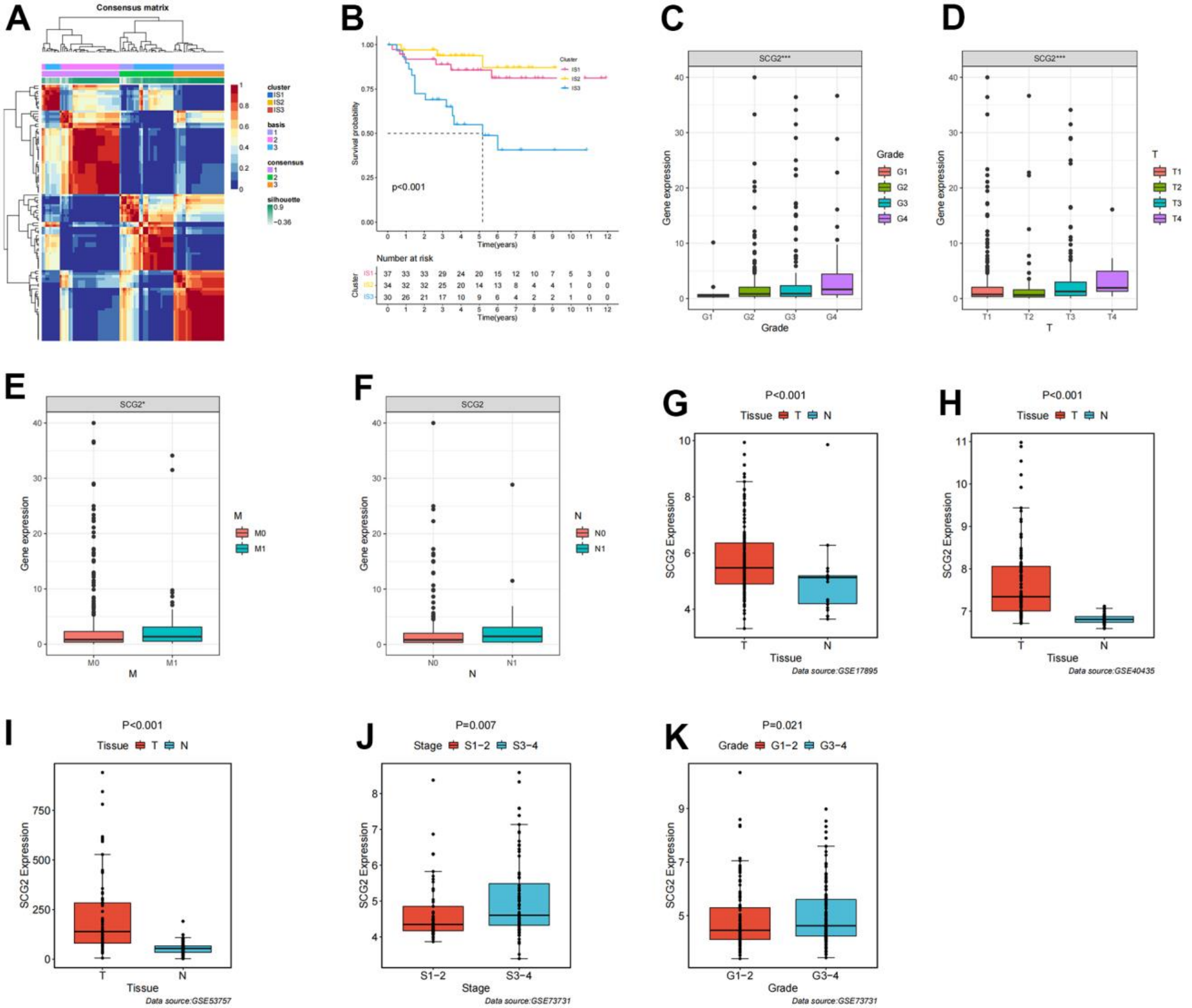


**Supplementary Figure 2. Identification of clinical characteristics of SCG2.** (**A**) Identification of three distinct ccRCC immune-related molecular subtypes in the E-MTAB-1980 dataset by the NMF algorithm. (**B**) Kaplan-Meier OS curve for three distinct ccRCC immune-related molecular subtypes. (**C**–**F**) Correlation analysis of SCG2 expression levels and different clinical features in the TCGA database. (**C**) Grade, (**D**) T stage, (**E**) M stage, (**F**) N stage. (**G**–**I**) Different expression of SCG2 in ccRCC tissues and normal tissues in the GEO database. (**G**) GSE17895, (**H**) GSE40435, (**I**) GSE53537. (**J**, **K**) Correlation analysis of SCG2 expression levels, histological grade, and pathological stage in the GSE73731 database.
